# Perioperative management of thoracoscopic left cardiac sympathetic denervation for refractory long QT syndrome: a case report

**DOI:** 10.1186/s40981-025-00815-7

**Published:** 2025-10-22

**Authors:** Asako Nitta, Atsushi Sawada, Kanami Abe, Naoyuki Kamiyama, Yuki Takahashi, Masahiro Miyajima, Mitsutaka Edanaga, Michiaki Yamakage

**Affiliations:** 1 Department of Anesthesiology, Sapporo Medical University School of Medicine, S1W16, Chuo-Ku, Sapporo, 060-8543 Japan; 2 Division of Anesthesia, Oji General Hospital, 3-4-8, Wakakusa-Cho, Tomakomai, 053-8506 Japan; 3 Department of Cardiovascular, Renal, and Metabolic Medicine, Sapporo Medical University School of Medicine, S1W16, Chuo-Ku, Sapporo, 060-8543 Japan; 4 Department of Thoracic Surgery, Sapporo Medical University School of Medicine, S1W16, Chuo-Ku, Sapporo, 060-8543 Japan

**Keywords:** Refractory long QT syndrome, Left cardiac sympathetic denervation, Stellate ganglion block, General anesthesia, Paravertebral block

## Abstract

**Background:**

Long QT syndrome (LQTS) refractory to standard treatments, including β-blockers and implantable cardioverter-defibrillators (ICDs), has been indicated for left cardiac sympathetic denervation (LCSD) in Europe and the United States. However, the clinical implementation of LCSD remains rarely performed in Japan as it is not covered by national health insurance.

**Case presentation:**

A 49-year-old woman with LQTS experienced frequent ICD activations, and β-blocker up-titration was limited due to severe heart failure. As a stellate ganglion block transiently shortened QT interval, LCSD was considered to prevent life-threatening arrhythmic events. Total intravenous anesthesia combined with a left thoracic paravertebral block was used to attenuate sympathetic nervous activation. Thoracoscopic LCSD was performed without arrhythmia or hemodynamic instability. The patient remained stable postoperatively, with no further ICD activations.

**Conclusion:**

This case demonstrates the safe perioperative management of LCSD for drug-refractory LQTS by incorporating strategies to minimize QT prolongation and suppress malignant arrhythmias.

## Background

Long QT syndrome (LQTS) is a congenital or acquired disorder characterized by impaired ventricular repolarization first described in 1957 [1], manifesting prolongation of QT interval, polymorphic ventricular tachycardia (VT) known as Torsades de Pointes (TdP), or ventricular fibrillation (VF). These abnormal electrocardiograms (ECGs) can present syncope and/or sudden arrhythmic cardiac death, which are life-threatening events among young populations. Standard treatments include pharmacological interventions, such as β-blocker, which is widely known to reduce cardiac events [2, 3]. However, some cases experience recurrent arrhythmias despite the treatment [3]. In such cases, left cardiac sympathetic denervation (LCSD) has been shown to effectively reduce cardiac events and has been performed in many countries [4]. However, in Japan, LCSD for refractory LQTS is not covered by national health insurance, and its clinical implementation remains extremely limited. Here, we report a case of refractory LQTS complicated by severe heart failure in which thoracoscopic LCSD was successfully performed, leading to suppression of life-threatening arrhythmias.

### Case presentation

**Fig. 1:**
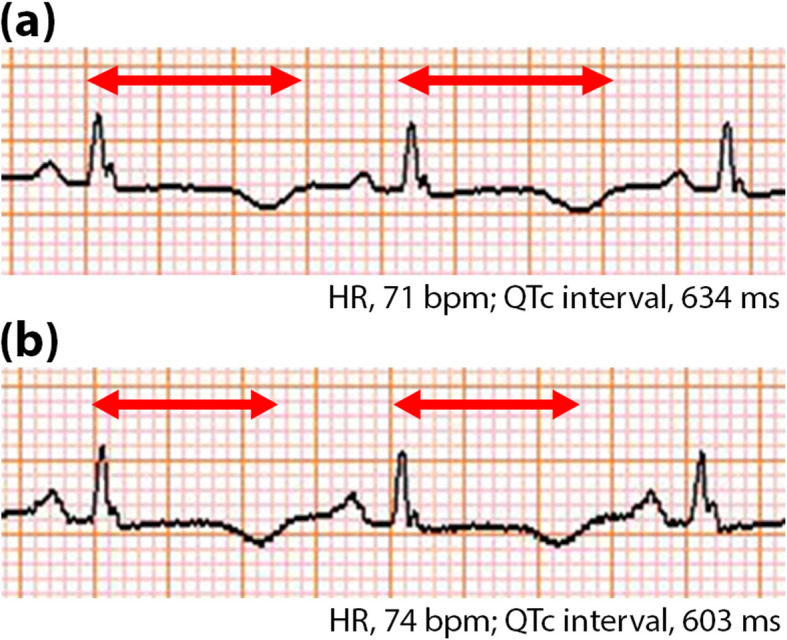
Stellate ganglion block transiently shortened corrected QT interval. Panels (**a**) and (**b**) show the electrocardiograms before and after the block, respectively. The red bidirectional arrows denote the duration of the corrected QT intervals. HR, heart rate; QTc, corrected QT interval

A 49-year-old woman (height, 155 cm; weight, 68.3 kg; body mass index [BMI], 28.4 kg**/**m^2^) was urgently transferred to a tertiary care hospital five years prior due to recurrent VT and VF occurring during hemodialysis for end-stage diabetic nephropathy. Genetic testing confirmed a diagnosis of congenital LQTS type 1. She was initiated on β-blocker therapy (bisoprolol 2.5 mg/day) and received an implantable cardioverter-defibrillator (ICD), after which she was discharged home. However, she subsequently experienced recurrent TdP and VF, with up to 114 ICD activations over 48 h. Despite attempts to up-titrate β-blocker therapy, she was readmitted due to cardiogenic shock resulting from severe heart failure, precluding further up-titration. The transthoracic echocardiography revealed an ejection fraction (EF) of 24.5%, moderate mitral regurgitation (MR), and a cardiac index (CI) of 1.3 L/min/m^2^. She required intra-aortic balloon pumping and catecholamine support, taking 1 month to achieve complete weaning.Her New York Heart Association functional class improved modestly from IV to Ⅲ, and cardiac function showed slight improvement with an EF of 32.3%, mild MR, and a CI of 2.0 L/min/m^2^. Given the recurrent life-threatening arrhythmias and the inability to up-titrate β-blocker therapy required for arrhythmia suppression owing to poorly controlled heart failure, there was concern that frequent ICD activations could trigger catecholaminergic storms, further exacerbating cardiac dysfunction. Therefore, stellate ganglion block (SGB) was considered for evaluating the efficacy of sympathetic nervous system inhibition. Under ultrasound guidance, 5 mL of 1% lidocaine was injected into the left longus colli muscle at the C6 transverse process level, judging the efficacy by the presence of transient Horner’s syndrome. A follow-up ECG performed 6 h later revealed a temporary and modest shortening of the corrected QT interval (QTc) from 634 to 603 ms (Fig. 1). According to the diagnostic criteria for congenital LQTS, the risk score based on the QTc is stratified within the range of 450–480 ms [5]. Although the QTc in this case exceeded this range, a 30-ms QTc shortening may have contributed to a potential decrease in the risk of cardiac events through sympathetic blockade. After obtaining the approval from the Department of Medical Safety in Sapporo Medical University Hospital, thoracoscopic LCSD was scheduled.

**Fig. 2:**
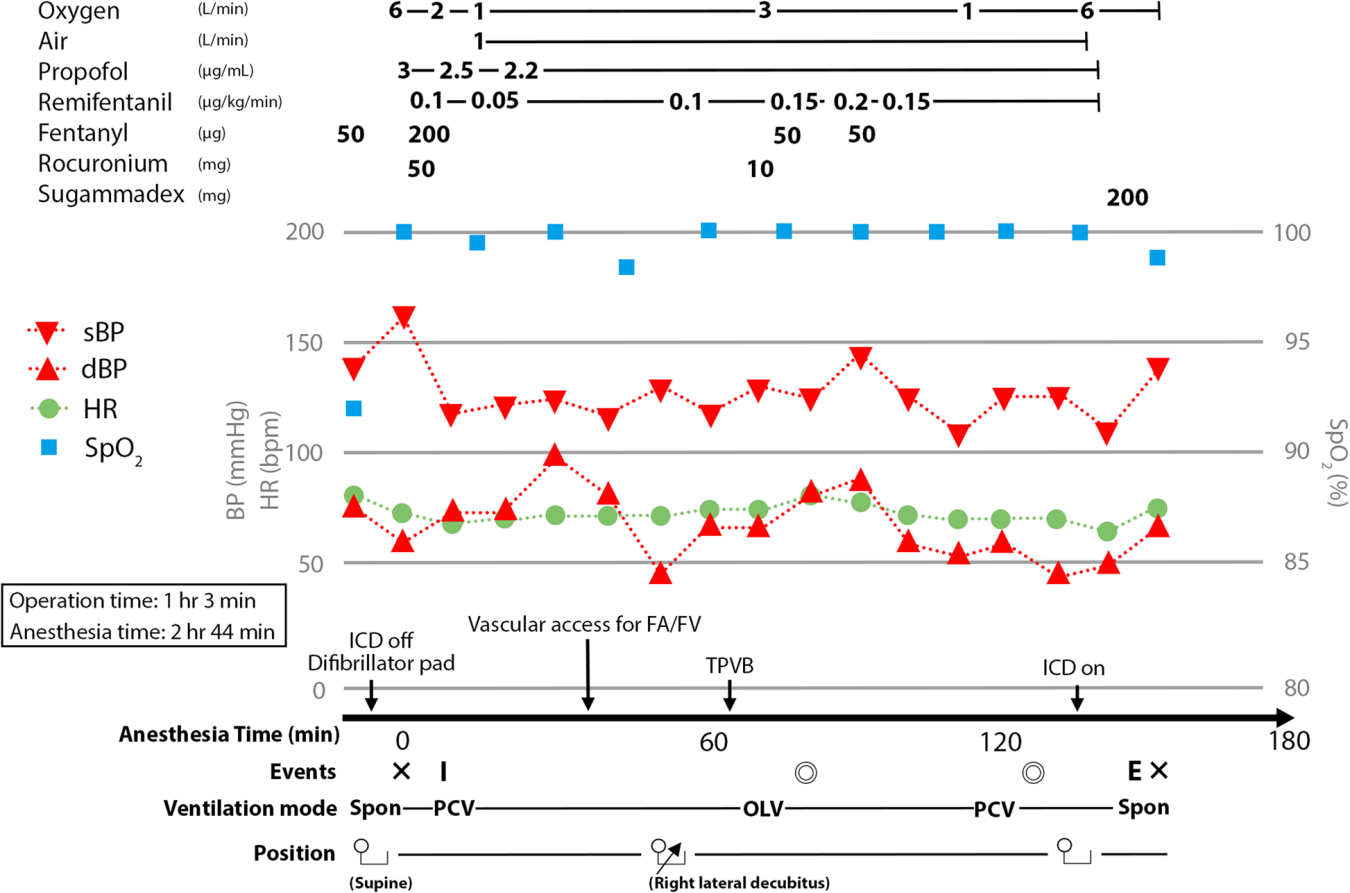
Anesthesia record of the present case. The LCSD procedure was completed without the occurrence of arrhythmias and without the need for the use of vasopressors, inotropic agents or mechanical circulatory support. sBP, systolic blood pressure; dBP, diastolic blood pressure, SpO_2_, percutaneous oxygen saturation; ICD, implantable cardioverter-defibrillators; FA, femoral artery; FV, femoral vein; TPVB, thoracic paravertebral block; Spon, spontaneous respiration; PCV, pressure controlled ventilation; OLV, one lung ventilation; ✕, start and end of anesthesia; ◎, start and end of surgery; I, tracheal intubation; E, tracheal extubation

**Fig. 3:**
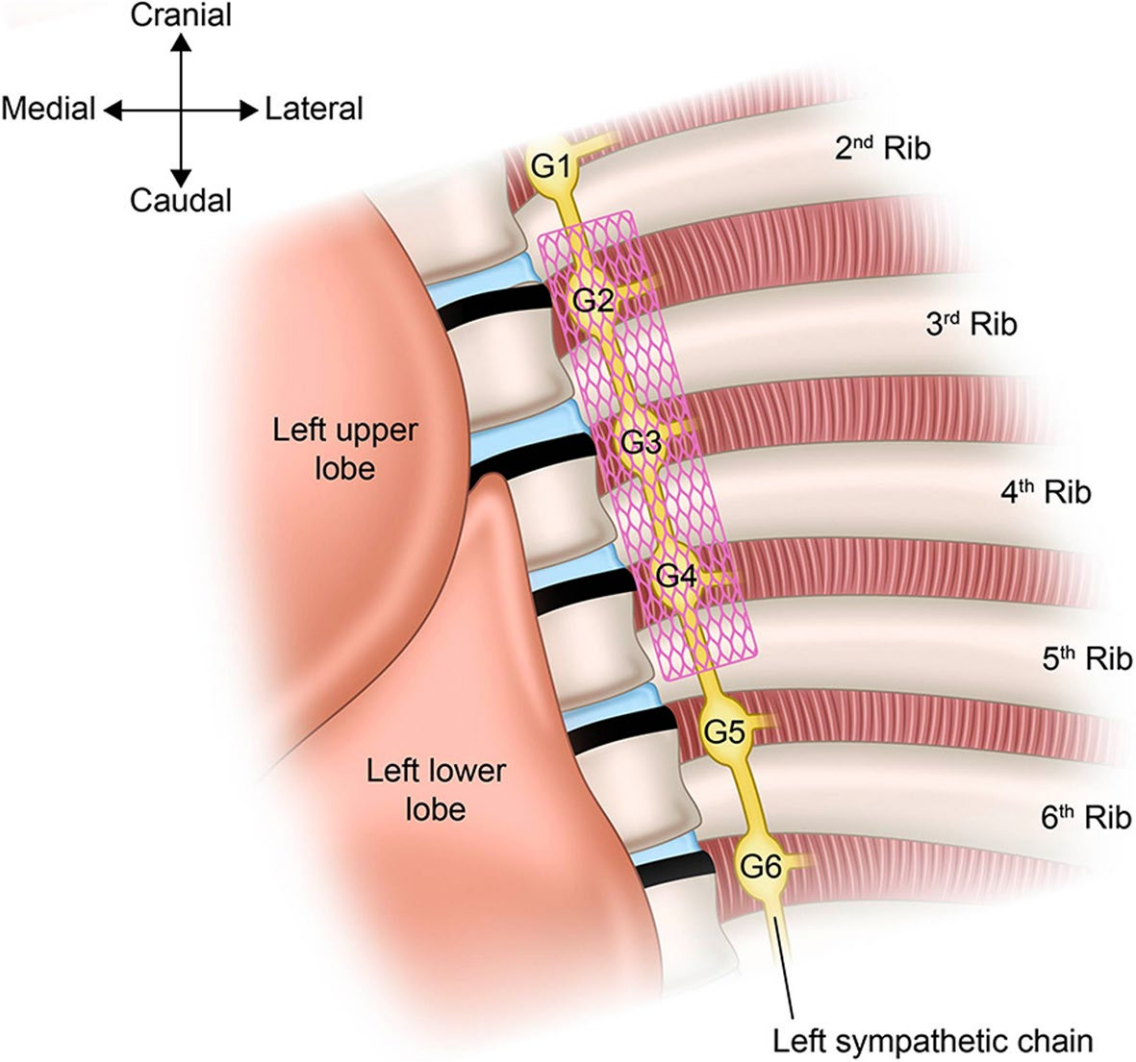
Schematic illustration of thoracoscopic LCSD. Following pleural incision over the sympathetic ganglia at the levels of the second, third, and fourth ribs, the exposed ganglia were cauterized, dissected, and resected. The pink-shaded rectangle indicates the extent of resected sympathetic ganglia. This illustration was created by Editage based on the original draft prepared by AN and YT. G, ganglion

The anesthetic management protocol on the day of surgery was as follows (Fig. 2). As she was in a stable mental state, no premedication was administered. The ICD was deactivated to prevent inappropriate activations associated with surgical manipulation, and transcutaneous defibrillator pads were applied. General anesthesia was induced with propofol at a target plasma concentration of 3 μg/mL using a Terumo® infusion pump equipped with Diprifusor software implementing the Marsh model, fentanyl 250 μg, remifentanil 0.1 μg/kg/min, and rocuronium 50 mg. Anesthesia was maintained with propofol 2.2 μg/mL, fentanyl 100 μg, remifentanil 0.05–0.2 μg/kg/min, rocuronium, and the left thoracic paravertebral block (TPVB). A double-lumen endotracheal tube was used for one-lung ventilation due to the thoracoscopic procedure. Given the preoperative risk evaluation, extracorporeal membrane oxygenation (ECMO) was to be initiated if vasopressor-refractory hypotension occurred or three or more defibrillations were required. Therefore, percutaneous vascular access was secured in the right femoral artery and vein. Then, she was positioned into the right lateral decubitus position. The TPVB was performed under ultrasound guidance, in an attempt to inhibit cardiac adverse events resulting from sympathetic activation by surgical manipulation before the completion of LCSD. Using an intercostal approach at T4 level, a single injection of 20 mL of 0.25% levobupivacaine was administered into the left paravertebral space. In video-assisted thoracoscopic LCSD, a single port was inserted through the third intercostal space at the level of the left anterior axillary line. Under thoracoscopic guidance, the left sympathetic ganglia aligned in the sagittal plane along the rib heads were identified. The ganglia were then resected from the upper border of T2 to the lower border of T4 (Fig. 3). No significant QT interval changes, arrhythmias, or hemodynamic instability were observed before or after ablation, and ECMO was not required. After LCSD was completed, the ICD was reactivated and she was immediately extubated in the operating room. Upon confirming stable respiratory and hemodynamic status, she was transferred to the intensive care unit for further monitoring. Although the QTc remained unchanged after LCSD (516 ms 2 days before surgery and 514 ms 4 days after surgery), the ICD did not activate throughout the postoperative period. No apparent complications, such as left-sided Horner’s syndrome and abnormal sweating, were observed. β-blocker up-titration was cautiously resumed to enhance the prevention of life-threatening arrhythmias. Additionally, she experienced muscle weakness due to prolonged bed rest, necessitating a considerable period of rehabilitation. She was discharged home on hospital day 74 since admission.

## Discussion

In LQTS, cardiac events are often exacerbated by sympathetic stimulation [6]. Therefore, suppressing this is a crucial strategy for sudden cardiac death. However, in some cases, the efficacy of β-blockers is limited, and frequent ICD discharges may further exacerbate sympathetic stimulation and ventricular arrhythmias, leading to a vicious cycle that necessitates additional shocks [7]. In such cases, LCSD has been suggested as a valuable therapeutic option. It has been reported that LCSD reduces annual cardiac adverse events by 86% [4]. The superiority of left over right cardiac sympathetic innervation in regulating ventricular catecholamine release provides a physiological basis for its efficacy of LCSD [8]. The 2017 American Heart Association guideline strongly recommends LCSD for refractory LQTS cases with ineffective or intolerable β-blocker therapy and frequent ICD discharges [9]. Notably, thoracoscopic sympathectomy is also established for palmar, axillary, and facial hyperhidrosis, involving bilateral excision of the sympathetic ganglia at T2, T3, or T4, depending on the location of sweating [10].

A previous case report documented the suppression of VT storms following SGB, which subsequently led to the decision to perform LCSD [11]. Given that SGB can be performed at the bedside, SGB may serve as a simple and practical tool for identifying candidates for LCSD prior to the invasive intervention. Even if SGB fails to shorten QTc or is not feasible due to factors such as antithrombotic medication, proceeding directly to LCSD is still reasonable when arrhythmias remain poorly controlled despite maximal medical therapy. One possible reason for the lack of QTc shortening following LCSD in this case is that the stellate ganglion was not included in the resection. Importantly, increased heterogeneity in ventricular repolarization is a key trigger of TdP, and QTc prolongation is not the sole electrophysiologic marker of risk [12]. Indeed, an increased VF threshold achieved by LCSD does not necessarily correlate with QTc shortening [13]. In this case, LCSD may have been sufficient to raise the threshold for malignant arrhythmias through decreasing the heterogeneity in ventricular repolarization. Anticipated side effects of LCSD—such as left-sided Horner’s syndrome and abnormal sweating, none of which were observed in this case—are generally regarded as acceptable, given the therapeutic goal of preventing life-threatening arrhythmias [14].

To date, no standardized anesthetic management guidelines exist for patients with LQTS. It is essential to minimize sympathetic nervous stimulation caused by pain and other stressors and to select anesthetic agents with a minimal risk of QT prolongation to reduce the likelihood of intraoperative TdP [15]. Intravenous anesthetics such as propofol are advantageous over volatile anesthetics because they have negligible effect on QTc duration at clinical doses and lower incidence of postoperative nausea and vomiting, thereby reducing the need for antiemetics, which themselves may prolong QT interval. Additionally, opioids such as fentanyl and remifentanil, as well as rocuronium, which have not been associated with QT prolongation, were administered. Adequate analgesia is also critical for suppressing deleterious sympathetic nervous system activity. A previous multicenter study demonstrated that epidural anesthesia reduces ventricular arrhythmias through sympathetic inhibition [16]. However, bilateral sympathetic blockade can lead to hypotension and circulatory suppression. In contrast, TPVB provides unilateral sympathetic blockade and is associated with less hemodynamic compromise [17], which was considered preferable in patients with severe heart failure. In this case, meticulous selection of anesthetic agents as well as pain management strategies enabled safe perioperative management without hemodynamic instability or life-threatening arrhythmias.

In conclusion, we describe a case in which perioperative management of thoracoscopic LCSD was successfully implemented for refractory LQTS complicated by the recurrent malignant arrhythmias.

## Data Availability

The datasets used during the current case are available from the corresponding author upon reasonable request.

## Abbreviations

LQTS: Long QT syndrome
ICD: Implantable cardioverter-defibrillators
LCSD: Left cardiac sympathetic denervation
VT: Ventricular tachycardia
TdP: Torsades de Pointes
VF: Ventricular fibrillation
ECG: Electrocardiogram
BMI: Body mass index
EF: Ejection fraction
MR: Mitral regurgitation
CI: Cardiac index
SGB: Stellate ganglion block
QTc: Corrected QT interval
ECMO: Extracorporeal membrane oxygenation
TPVB: Thoracic paravertebral block
